# Analysis of Anaphylactic Shock Caused by 17 Types of Traditional Chinese Medicine Injections Used to Treat Cardiovascular and Cerebrovascular Diseases

**DOI:** 10.1155/2015/420607

**Published:** 2015-04-27

**Authors:** Yu-Jiao Guo, De-Wang Wang, Ling Meng, Yong-Qing Wang

**Affiliations:** Department of Pharmacology, The First Affiliate Hospital of Nanjing Medical University, Nanjing, Jiangsu, China

## Abstract

Several reports describing anaphylactic shock following treatment of cardiovascular and cerebrovascular diseases with Chinese herbal injections were described. Our analysis of these reports showed that anaphylactic shock caused by traditional Chinese medicine (TCM) injections for the treatment of cardiovascular and cerebrovascular diseases is common but also sometimes fatal. Therefore, we proposed the following four suggestions for improving the clinical safety of delivering Chinese herbal injections and reducing the occurrence of allergic shock. First, patients with cardiovascular and cerebrovascular diseases are at high risk, so they should only be given TCM injections after a doctor's diagnosis and approval. Second, people in allergic groups can suffer anaphylactic shock, so vigilance is important in the treatment of all age groups, although even more caution should be exercised when treating children or elderly people. In fact, TCM injections may not be appropriate for those age groups, so that they should be carefully considered before treatment. Third, no significant gender differences have been noted in patients with anaphylactic shock, so all patients should be carefully monitored, irrespective of gender. Fourth, the timeframe in which different drugs cause anaphylactic shock varies; thus, patients should be observed as long as possible.

## 1. Introduction

In recent years, traditional Chinese medicine (TCM) injections have been widely used in the clinic for the treatment of many conditions, including hypertension, coronary heart disease, diabetes, nephrosis syndrome, rheumatoid arthritis, fracture, and cervical degenerative disease. However, a new dosage form of TCM injections has changed the traditional route of administration, while still retaining the characteristics of TCM. This new form works more quickly and effectively in treating certain disease [1], especially for cardiovascular and cerebrovascular disease, digestive system disease, respiratory system disease, tumor, and so on. It is found that compound Danshen, Honghua, Shuxuetong, Ginkgo biloba, ligustrazine,* Erigeron breviscapus*, Ciwujia, Mailuoning, Ge Gensu, and Dan Hong injection have obvious advantages in the treatment of coronary heart disease, angina, and acute cerebral infarction [2]. Recently, with the increased incidence of cardiovascular and cerebrovascular diseases, the usage of TCM injections has increased each year due to its clinical efficacy. However, despite the effectiveness of TCM injections in treating some diseases, its toxicity has been of concern as it can induce adverse drug reactions (ADRs), such as allergy including anaphylactic shock, a common side effect. Therefore, the toxicity of TCM injections should be recognized and, as such, be carefully utilized.

Allergy occurs when the immune system develops specific antibodies against an exogenous antigen (allergen) that results in an exaggerated response toward a substance that is normally harmless, thereby causing tissue damage. Anaphylactic shock is a severe, systemic allergic reaction to a specific allergen that occurs due to acute peripheral circulatory failure. It is rapid in onset and can be fatal if not treated quickly. The clinical manifestations of anaphylactic shock include heart palpitations, chest tightness, laryngeal obstruction, dyspnea, pale or cyanotic complexion, chills, sweating, cold perception in the limbs, weak pulse, drop in blood pressure, loss of consciousness, coma, convulsions, incontinence, and even sudden cardiac death. China's National Center for Adverse Drug Reaction listed the top 10 TCM injections that caused serious side effects. Of these, seven types were responsible for the most cases of anaphylactic shock and included Shenmai injection, Xuesaitong injection,* Salvia miltiorrhiza* injection, compound Danshen injection, Shengmai injection, Xueshuantong injection, and Mailuoning injection. Mailuoning injection caused 64 cases of anaphylactic shock. In addition, between January 1, 2011 and December 31, 2011, the National Drug Adverse Reaction Monitoring Center received a total of 1500 ADR case reports from Mailuoning injection, of which 189 cases were severe. Compound Danshen injection caused 53 cases of anaphylactic shock. On March 24, 2009, the Ministry of Health and China's State Food and Drug Administration jointly issued an urgent notice requesting the immediate termination of the usage, selling, and production of compound Danshen injection from Taizhou Tianrui Pharmaceutical Company. Ciwujia injection caused 33 cases of anaphylactic shock, with the most severe cases occurring after October 5, 2008. Subsequent investigation showed that these serious ADRs were caused by drug contamination.

Since these drugs have similar pharmacological effects, their common characteristics may cause ADRs. However, these injections also have specificity, as they have distinct chemical compositions [1]. In 2009, the article has reported serious anaphylaxis caused by nine Chinese herbal injections used to treat common colds and upper respiratory tract infections [3]. Also, another article has reported anaphylactic shock and lethal anaphylaxis caused by* Houttuynia cordata* injection for antibacterial and antiviral therapy [4]. So far, there is no study on anaphylactic shock caused by traditional Chinese medicine injections used to treat cardiovascular and cerebrovascular diseases. In order to further explore the characteristics underlying the ADRs to TCM injections and to provide evidence that may ensure the safe and effective clinical usage of TCM injections, we studied the general pattern and characteristics of anaphylactic shock caused by TCM injections.

## 2. Cases

In this report we analyzed 316 articles, collected from medical journals published in China between 1980 and 2013 that described cases of anaphylactic shock caused by herbal injections for cardiovascular and cerebrovascular diseases. A total of 17 different types of herbal injections were described in these reports. Collectively, 350 episodes of anaphylactic shock and 10 deaths were reported (summarized in Table 1). All 10 lethal anaphylaxis incidences occurred following intravenous injection (IVI). Among the 350 anaphylactic shock cases, 4 patients (1.14%) were administered intramuscular injections (IMI), and 346 patients (98.86%) were given IVI. Patient age ranged from 9 to 97 years. Of the 350 patients who suffered anaphylactic shock, 5 were children under the age of 18, 108 were between 19 and 45 years of age, 104 were between 46 and 59 years of age, 132 were over 60 years of age, and 1 patient was a pregnant woman. The studies included a total of 169 females and 181 males. The interval between herbal injection and time of anaphylactic shock ranged from 3 s to 2.5 h. The patients in these studies were those who were hospitalized for cardiovascular and cerebrovascular diseases. When allergic reactions to herbal injections occurred, all patients were immediately taken to the emergency department (Table 1).

## 3. Analysis

### 3.1. Shenmai Injection

Shenmai injection can be used for the treatment of shock, coronary heart disease, viral myocarditis, chronic cor pulmonale, and neutrophils to reduce. It also can improve the immune function of tumor patients and reduce the adverse reaction caused by chemotherapy. Shenmai injection mainly contains ginseng,* Ophiopogon japonicus*, the active components of ginseng saponin, flavones, and trace ginseng polysaccharides. These components can stimulate the body to produce antibodies, resulting in adverse ADRs. In addition, the improper use of red ginseng can cause severe ADRs, such as psychiatric and neurological symptoms, arrhythmia, gastrointestinal bleeding, or even death [5]. It is possible that the pathogenesis of red ginseng is due to component structural changes and modifications that occur after preparation of red ginseng and* Ophiopogon japonicas*; however, further studies are needed to validate this theory. The content of* Ophiopogon japonicus* in Shenmai injection is significantly lower than that of* Panax* species, so more studies have focused on ginseng (species of the genus* Panax*). Ginsenosides Rb1, Rb2, Rc, Rd, Re, Rg1, and* Ophiopogon in* D are the active and main components of Shenmai injection. Test results after Shenmai injection showed that the blood concentration of ginsenoside Rb1 was relatively high, but those of ginsenoside Rg1 and ginsenoside Re were low. Ginsenosides Rg1 and Re were rapidly distributed and eliminated* in vivo*, but ginsenoside Rb1 was slowly metabolized* in vivo*. The efficacy of ginsenoside Rb1 may be related to the fact that it has a long half-life of up to 47 h [6], so the anaphylactic shock caused by Shenmai injection may be mainly due to ginsenoside Rb1.

### 3.2. Ciwujia Injection

Ciwujia injection contains several active components, including Eleutheroside, isofraxidin glycosides, Ding Xiangdai, Hyperoside, and* Acanthopanax senticosus* polysaccharide. These components can dilate blood vessels, increase coronary blood flow, increase myocardial oxygen consumption, improve blood circulation, and increase appetite. It has been difficult to determine the main component that causes anaphylactic shock, and it may be caused by the active components themselves or impurities in the drug preparation.

As aforementioned, anaphylactic shock is a serious, potentially life-threatening allergic response. Ciwujia injection contains various active polymers, and once administered directly into the blood stream intravenously, this exogenous antigen stimulates the immune system and causes an allergic reaction. No significant relationship has been found between Type I allergic reactions and drug concentration and dosage, indicating that allergic reactions are associated with drug quality and the active components or impurities in the drug preparation [7]. It has been reported that patients with drug allergies are more susceptible to anaphylactic shock from Ciwujia injection; thus, before treatment, patients should be asked to detail their history of drug sensitivity [8]. In addition, patients with idiosyncratic reactions should be banned from taking this drug [9].

### 3.3. *Salvia miltiorrhiza* (Danshen) Injection

*Salvia miltiorrhiza* can dilate coronary artery, increase the blood volume, improve myocardial ischemia, promote myocardial ischemia or injury recovery, and reduce the myocardial infarction scope. It also can fight against thrombosis, regulate blood lipid, inhibit the formation of atherosclerotic plaques, and protect liver cells and gastric mucosa. It also has anti-inflammatory and antiallergic effect. The mechanism underlying the allergic reaction to* Salvia miltiorrhiza* injection is unclear; however, there are two possibilities. First, tanshinone and acid crystals in the* Salvia miltiorrhiza* injection may bind to plasma proteins and have sufficient immunogenicity to cause an allergic reaction. Second, the active components of* Salvia miltiorrhiza* injection are caffeic acid ester derivatives that polymerize to form orthodihydroxy compounds. It is similar to the structure of tannin and also binds to plasma proteins [10]. It has been reported that the anaphylactic shock caused by* Salvia miltiorrhiza* injection was related to the drug concentration. Specifically, two cases [11] of anaphylaxis occurred upon administration of 24 g* Salvia miltiorrhiza* using 250 mL low molecular weight dextran for an intravenous drip at 30 drops/min. However, when the* Salvia miltiorrhiza* concentration decreased to 10 g, no cases of anaphylactic shock occurred. Therefore, it appears that allergic shock was caused by the high density of the drug.* Salvia miltiorrhiza* can slow the heart rate and dilate blood vessels. At high concentrations, excessive doses, and fast drip rates, its concentration in the bloodstream increases rapidly to dilate small blood vessels and decrease blood pressure. Therefore, in the clinic, input concentration, velocity, and dose of* Salvia miltiorrhiza* should be tightly controlled, particularly in patients with bradycardia, to avoid the occurrence of severe ADRs [12]. In addition,* Salvia miltiorrhiza* combined with low molecular weight dextran has a larger probability of causing anaphylactic shock. Low molecular weight dextran functions as a blood volume expander but also has mild anticoagulant effects. At the same time,* Salvia miltiorrhiza* can promote blood circulation to remove blood stasis and increase the number of mast cells. After combining* Salvia miltiorrhiza* with low molecular weight dextran, the extracellular fluid moves from the blood vessels into the tissue, and the mast cells release chemical mediators such as histamine and serotonin. These chemical mediators can cause muscle spasms and increase vascular permeability. Furthermore, the antigenicity of dextran can stimulate the body to produce antibodies, resulting in anaphylactic shock. The residual ethanol in the Danshen injection can undermine the dextran precipitates and destroy colloidal solution, causing drug degeneration. Thus,* Salvia miltiorrhiza* should not be combined with low molecular weight dextran for intravenous administration [13]. The same to Danhong injection and Compound Danshen injection those contain Danshen.

### 3.4. Tanshinone IIA Sodium Sulfonate Injection

Tanshinone IIA is derived by sulfonation of the water-soluble substance Tanshinone IIA sulfonate. Tanshinone IIA is derived from phenanthrene-quinone, which is isolated from* Salvia miltiorrhiza*. Tanshinone IIA sodium sulfonate can increase coronary flow, inhibit platelet aggregation and antithrombotic ischemia, reduce myocardial infarct size, and so on [14]. Tanshinone IIA increases water solubility by sulfonation, so Tanshinone IIA sodium sulfonate has high solubility in water. However, because Tanshinone IIA sodium sulfonate is susceptible to hydrolysis, the soluble components are separated from the insoluble ones; therefore, Tanshinone IIA sodium sulfonate injection has slight precipitate particles during long-term storage that can cause allergic reactions in the body. In fact, reports have shown that anaphylactic shock is caused by the small amount of impurities and resin remnants that remain after the extraction process.

### 3.5. Danhong Injection

Danhong injection can be used for antiatherosclerosis, inhibit platelet aggregation, and protect vascular endothelial cells. The main components of Danhong injection are* Salvia miltiorrhiza* and safflower. The mechanism by which Danhong injection induces anaphylactic shock is similar to that of* Salvia miltiorrhiza*. Safflower injection may contain pollen protein, which can cause anaphylactic shock [10]. Because Danhong injection contains* Salvia miltiorrhiza*, it should also not be combined with low molecular weight dextran. This was proven when a patient in one case report suddenly succumbed to anaphylaxis after being administered an intravenous drip of Danhong combined with 250 mL low molecular weight dextran [15]. Danhong injection has certain drip specifications. For example, patients with heart conditions can receive Danhong injection at a rate of ~30–40 drops/min, but for other adults, an injection rate of 60 drops/min is suitable; the injection rate for children varies according to age and physical circumstances. The intravenous drip rate can greatly affect the incidence of ADRs, so the doctor should carefully control drip speed and closely observe the patient's reaction. Danhong injection has a safe medication dose range and must be used in accordance with the drug dosage given in the instructions. Patients can take Danghong injection at ~20–30 drops/min for 1-2 times per day. Danghong should be diluted with 5% glucose (100–500 mL) for injection. Some patients with allergic shock may be related with solvent. Yuan and Zhao [10] reported that one patient, aged 83, due to the eyes of ischemic optic nerve disease in hospital, with a history of hypertension, cerebral infarction, and myocardial infarction, with no history of adverse drug reaction, suffered anaphylactic shock when he/she accepted Dan Hong injection 20 mL with 0.9% NS 250 mL intravenous drip on day 8. Therefore, except special condition, the user of Danhong injection should be strictly in accordance with the drug instructions.

### 3.6. Breviscapine Injection

Breviscapine injection contains flavonoids, scutellarin, and the* Erigeron* 60 hormone, although scutellarin is the main component. Scutellarin can inhibit the intrinsic coagulation system, promote the activity of plasmin, reduce platelet count, and inhibit platelet aggregation. Flavonoids in acidic environments are easy to precipitate; therefore, breviscapine is stable in 0.9% NaCl or 5% glucose for injection. In addition, the pH should not be less than 4.2 or it will crystallize, leading to an increase in the number of particles and subsequent allergic reactions. Breviscapine injection is a pure TCM preparation with a complex composition.

### 3.7. *Erigeron* Injection

*Erigeron* injection is composed of* Erigeron breviscapus* extracted with sterile water solution made of phenolic compounds.* Erigeron* injection esters mainly contain scutellarin and coffee. It is commonly used for the treatment of ischemic stroke, coronary heart disease, and angina pectoris due to the fact that it promotes circulation and removes stasis. The literature has reported that* Erigeron breviscapus* solution is weakly alkaline (pH 7.0–7.5). When the pH is low,* Erigeron breviscapus* solution can easily crystallize, thereby increasing the number of insoluble particles. In addition, when glucose is used as the solvent for* Erigeron breviscapus* injection, there is an increase in the number of insoluble particles, whereas the number of particles is much lower when 0.9% NaCl is used as the solvent. In these studies, 5% glucose was used as the solvent in most patients, although the instructions stated that the solvent should be 0.9% NaCl. He and Lei [16, 17] reported that three patients with the use of 10% glucose or 5% glucose as solvent showed the symptoms of anaphylactic shock.

The insoluble particles may be the main cause of allergic shock, so 0.9% NaCl should be used as the solvent in the clinic. Although there did not appear to be a significant correlation between drug dose and anaphylactic shock, some patients were injected with up to 100 mL* Erigeron breviscapus*, which is two times more than the instructions specified. In particular, elderly people had poor tolerance and were more likely to suffer from ADRs.

### 3.8. Compound Danshen Injection

Compound Danshen injection is composed of Danshen extract and* Lignum dalbergiae* odoriferae extract.* Salvia miltiorrhiza* can scavenge free radicals and resist lipid peroxidation;* Dalbergia *can reduce lipid peroxidation injury.* Dalbergia* and* Salvia miltiorrhiza* have synergistic effects and can decrease heart rate, act as a sedative, and cause hypnotic and transient hypotension. The mechanism underlying allergic reactions induced by* Salvia miltiorrhiza* is similar to that of compound Danshen. Long-term toxicity studies of* Salvia miltiorrhiza* have shown that if it is infused too quickly, a fall in blood pressure will occur, which can easily cause anaphylactic shock [18]. Compound Danshen has an injection pH range of 4 to 6.5. The number of insoluble particles greatly increased when compound Danshen was mixed with 0.9% NaCl for injection at an alkaline pH. This increases the chance that a patient will suffer from an ADR. Most patients infused at a drip speed of 85 drops/min suffered from anaphylactic shock, which often occurred as quickly as a few seconds after injection. Thus, aninfusion rate of 30 drops/min is more appropriate. Importantly, patients should be closely observed for at least 15 min after injection, as patients cannot adjust the drip speed themselves. If the patient suffers from an ADR, the nurse should immediately close the valve and report to the physician. Compound Danshen causes mild vasodilatation; if the concentration is too high or if it is infused too quickly, the blood concentration of the drug will increase rapidly in a short period of time, causing blood vessel dilatation due to small decreases in blood pressure and bradycardia, which can lead to anaphylactic shock. Therefore, the input velocity and drug concentration must be tightly controlled with compound Danshen injection [19].

### 3.9. Puerarin Injection

Puerarin injection can dilate coronary and cerebral blood vessels, improve myocardial systolic function, inhibit platelet aggregation, reduce blood viscosity, and improve microcirculation. Anaphylactic shock mostly occurred in patients who were continuously using Puerarin, which belongs to I type allergic reaction. The patients recovered a few days after drug treatment was terminated and they received antiallergy medication. Anaphylactic shock may have been caused from impure substances in Puerarin resulting from drug preparation, which induced antigenicity [20].

Additionally, Puerarin itself or its byproduct may have decomposed by drug metabolism and then may have combined with a protein carrier to form a hapten carrier complex, resulting in an immune response. Puerarin injection mainly contains flavonoid glycosides but also contains small amounts of daidzein, daidzin, and other effective components of TCM. These components or impurities in the formulation can serve as antigens or haptens and cause anaphylactic shock. Puerarin is an isoflavone compound with low solubility. Isoflavones as strong planarity molecular are arranged closely between the molecule and another one, so it is difficult to dissolve in water. In order to increase the solubility of Puerarin, 50% propylene glycol was added to the injection as a solvent. Propylene glycol can degrade to produce pyruvic acid, lactic acid, acetic acid, and other allergens in certain conditions, which can cause vascular stimulation in patients and symptoms of fever. In addition, propylene glycol can directly cause the dissolution of red blood cells. Studies have shown that the number of particles in TCM injections is significantly greater than that in Western medicine intravenous injections. Puerarin injection, as a TCM preparation, may produce insoluble particles that cannot be metabolized in the body, resulting in allergic reactions [21].

If Puerarin injection is used as a treatment over a long period of time, toxic effects can be caused by drug accumulation. Most cases of anaphylactic shock resulted from the continuous use of Puerarin, unlike the anaphylactic shock caused by penicillin. Most people who suffered from an allergic reaction had no history of drug allergies; however, doctors should still ask patients to detail their personal and family histories of drug allergies before treatment, and patients with drug allergies should use Puerarin carefully. Patients who suffered from an ADR following Puerarin injection recovered within a few days of drug withdrawal and antiallergy medication. Before allergic shock occurred, patients had adverse reactions such as fever and chest tightness. When this occurs, doctors should immediately terminate the drug and treat the patients' symptoms.

The use of Puerarin should be strictly controlled, especially in elderly patients. Patients' blood routine, liver, and kidney function should be monitored, and treatment should not be for too long as the intermittent use of Puerarin can easily induce the production of antibodies. Large doses of medication can trigger immune complexes to cause tissue injury, and subsequent use of the medication will cause memory cells to respond quickly, leading to serious ADRs. Sun [22] reported that one patient, aged 58, with coronary heart disease, showed suddenly palpitations, shortness of breath, sweating, cold extremities, and other symptoms of anaphylactic shock 30 minutes after the intravenous drip of Puerarin on day 8. Therefore, we suggest a treatment schedule of 1 course for 1 week, which should be repeated after 1 week. This course of treatment will significantly reduce the risk of ADRs. Since Puerarin is a vasodilator, the patient's full medical history should be taken into account before it is administered [23].

### 3.10. Safflower Injection

Safflower injection is a TCM injection of safflower extract. It has many functions, such as scavenging free oxygen radicals, inhibiting platelet aggregation, and reducing vascular resistance and dilation of the coronary artery. Previous studies indicated that the excessive use of safflower injection can increase the number of insoluble particles, which can result in anaphylactic shock. Excessive use can also increase the liquid concentration, such that, at the same drip speed, patients are more susceptible to ADR due to the increase in pharmacological effects. The drug instructions recommend using 5% and 10% glucose (~250–500 mL) as the dilution solvent. Lu and Ye [24] reported that one patient, aged 63, suffering lumbar disc herniation, with history of cephalosporin allergy, appeared dizziness, chest tightness, shortness of breath, clammy skin, and other symptoms of anaphylactic shock when accepting intravenous injection of safflower injection +0.9% Sodium Chloride Injection 10 minutes later. In the first medication, compatibility and stability tests showed that as the pH of the solvent increased, the number of insoluble particles significantly increased as well. Thus, it is better to not combine safflower with 0.9% NaCl for injection [25].

ADRs induced by safflower injection involve multiple systems of the human body. Foreign antigens combine with antibodies in the material, leading to an abnormal immune reaction that may be due to the safflower injection or due to patients' specific allergies. The elements of safflower injection are complex and contain Safflor yellow and safflower glucoside. Antigens or haptens injected or transfused directly into the bloodstream can cause allergic reactions. In particular, when yellow pigment combines with sugar in the body, it forms a semiantigen. Hapten combines with the material in the red blood cell surface to form an antigen. Then, the antigen acts on the mast cells of target cells (laryngeal, tracheal, and bronchial) to produce IgE antibodies to cause allergic reactions. Some patients previously used safflower and produced specific antibodies in their bodies. In those cases, the intravenous drip of safflower injection acted as an allergen that activated the intracellular enzyme release of active substances, such as histamine like, causing an allergic reaction [26].

Safflower injection may contain pollen protein, which can cause anaphylactic shock. Therefore, we advised manufacturers to improve the purification process of safflower injection and try to remove impurities and pollen protein [27].

### 3.11. Mailuoning Injection

Mailuoning injection is a compound preparation that contains* Achyranthes* root, Radix Scrophulariae,* Dendrobium*, and honeysuckle. Mailuoning functions to clear away heat, nourish Yin, promote blood circulation, and remove blood stasis. The main components of honeysuckle are chlorogenic acid and isochlorogenic acid. Chlorogenic acid is an allergen that can cause allergic reactions, although it does not cause allergic reactions when being orally prepared [28].* Achyranthes*, Radix Scrophulariae,* Dendrobium*, and honeysuckle in Mailuoning injection are from nature. People who had contact with or used these drugs previously will be sensitive to them. Therefore, the majority of allergic reactions will occur on first drug use [29]. Mailuoning injection is prepared by chemical extraction; however, due to extraction methods, its purity is not guaranteed. In addition, during the preparation process, manufacturers add solubilizing agent, stabilizer, and other additives to improve the solubility and stability of the active components of Mailuoning injection. These additives can cause allergic reactions. For example, the use of polysorbate 80 as a solubilizing agent for TCM injections correlated with the occurrence and severity of ADRs.

### 3.12. Shengmai Injection

Shengmai injection contains ginseng,* Ophiopogon japonicus*, and* Schisandra chinensis*. Red ginseng is a type of ginseng, with ginsenoside being as the active component. Ginsenoside can adjust blood pressure, improve circulation, and promote metabolism and protein synthesis. The active component of* Ophiopogon japonicus* is* Maidong saponin*. The active component of Fructus Schisandra is Schisandrin. The effect of Shengmai injection is determined by the interaction of the 3 components, and there are many reasons that it can lead to ADRs.

Some possible reasons that Shengmai injection may cause anaphylactic shock include the fact that the treating physician may not fully understand the indications of Shengmai injection. For example, fracture of diaphragm fibers can be caused by Shengmai injection. Second, the physician may not ask patients about their personal and family history of drug allergies before administering the drug. This will cause some patients with drug allergies, food allergies, or chronic bronchial asthma to receive Shengmai injection, leading to ADRs. Third, some doctors may give patients an excessive dose of medication. The usage and dosage of Shengmai injection are specified as follows: 1 intravenous drip of 20–60 mL diluted with 5% glucose (250–500 mL). However, in some cases, the doctors gave an intravenous drip of 80–100 mL, which is more than the prescribed maximum dose of 60 mL. Fourth, the doctor may choose an inappropriate infusion solvent, which could lead to anaphylactic shock, although this theory needs to be further researched. Lastly, if Shengmai is infused too quickly, anaphylactic shock can occur [30]. Zhang [31] reported that 3 patients by intravenous of Shengmai injection 100 mL + 0.9% Sodium Chloride Solution 250 mL showed allergic shock symptoms in 5 minutes.

Since the majority of patients suffered allergic shock for the first time after Shengmai injection, and the majority of cases (13/16, 81.25%) occurred within 10 min after administration, we can conclude that allergic shock induced by Shengmai injection leads to an immediate allergic reaction. However, the components that cause anaphylactic shock are still unclear, although we speculate that ginsenoside may be the underlying culprit [32]. Only red ginseng Shengmai injection, all the effective components were determined. The effective components of* Ophiopogon japonicus, Schisandra*, have no quantitative determination. The quality of TCM injection cannot be guaranteed due to imperfect quality standards. Thus, additional studies are needed to strengthen the quality standard and to establish a method for determining the active components of specific drugs. This would most likely reduce the occurrence of ADRs.

### 3.13. Shuxuetong Injection

Extracts of the leech and earthworm are the active components of Shuxuetong injection. These extracts contain hirudin, earthworms, earthworm enzyme, and other antithrombotic substances. Hirudin is found so far the strongest thrombin specific inhibitor. It can reduce the activity of thrombin, block the formation of fibrin, prevent hemostatic response and platelet activation response induced by thrombin, and reduce platelet activity, exerting its anticoagulant effect. This enzymatic system expresses the tissue type plasminogen activator, which has strong fibrinolytic activity. Thus, ADRs may be associated with these protein components [33]. To prepare Shuxuetong injection, large molecules, such as amino acids, peptides, and mucopolysaccharides, are removed using a filter [34]. The molecular weight of the remaining polysaccharides, polypeptides, and amino acids with physiological activity is about 5800 Da and should be less than 1% of the Shuxuetong injection material. However, small amounts of residual polymer protein may cause ADRs [35]. Although the mechanism by which ADRs occur following Shuxuetong injection remains unclear, there are two characteristic possibilities. First, the protein constituents contained in leech and earthworm may have sensitizing effects. Second, Shuxuetong injection has anticoagulant and antithrombotic pharmacological activities and improves blood rheology. Improvements in technology may lead to improvements in drug purity, thereby reducing the occurrence of ADRs [36].

### 3.14. Xingnaojing Injection

Xingnaojing injection has detoxicating, cooling blood, and restoring consciousness effect. Xingnaojing injection is composed of musk, borneol, turmeric, and gardenia. Protein, fatty acids, and other molecules can be used as antigens in the body to stimulate the immune system to produce antibodies. The antibody attaches to mast cells, which then become sensitized. When mast cells are exposed to the same antigen, the antigen reacts with antibodies on the surface of mast cells, after which the mast cells release particles into the surrounding media [37]. Stimulation of the immune system can lead to an allergic reaction. The blood capillaries expand and their permeability increases, and the respiratory system, cardiovascular system, and skin and mucous membranes change to induce anaphylactic shock. The possibility of allergies induced by musk is great, possibly because musk is animal drugs [38].

### 3.15. Xuebijing Injection

Xuebijing injection mainly contains safflower, chuanxiong, and* Salvia*. Xuebijing injection can improve circulation, reduce infection and injuries induced by bacterial endotoxin, reduce the inflammatory response, and inhibit the formation of granuloma. During the process of extraction, there are many problems such as low purity and a large amount of residue. At the same time, the active components of safflor yellow A can enter the bloodstream and stimulate the body to produce antibodies or sensitized lymphocytes. Thus, when the allergen enters the body again, an allergic reaction will occur [39].

Manufacturers need to further improve the production process and quality of their products to strengthen the quality control of Xuebijing injection, which will reduce the occurrence of ADRs. The body is in a state of stress after an operation. The number of peripheral blood phagocytes increases, enhancing the immune system. This makes the body susceptible to allergic reactions.

### 3.16. Xuesaitong Injection

Xuesaitong injection is composed of panaxnoto ginseng extracted from* Panax pseudoginseng*. It can dilate cerebral vessels, inhibit platelet aggregation, and have antiatherosclerosis, antithrombosis, and antiarrhythmia activities. The elements of* Panax notoginseng* are complex, containing more than 20 kinds of saponin-active material and 17 kinds of trace elements, protein, vitamins, and polysaccharides.

There are four other possible causes of allergic reactions due to Xuesaitong injection. First,* Panax notoginseng* is the main component of Xuesaitong. It is not stable in aqueous solution, easily precipitates when stored, and directly affects the quality of the drug. Second, the dilution solvent for Xuesaitong is ethanol, so patients with alcohol allergies are banned from taking Xuesaitong injection. Third, Xuesaitong mixed with NaCl forms insoluble particles. Fourth, the occurrence of allergic shock correlates with the condition of the patient [40].

### 3.17. Xueshuantong Injection

Similar to Xuesaitong injection, Xueshuantong injection is composed of* Panax notoginseng* extracted from* Panax pseudoginseng*, with the main ingredients of ginsenoside Rg1 and Rb1. It can promote blood circulation, remove blood stasis, expand blood vessels, and improve microcirculation. The ADRs caused by Xueshuantong injection may be due to some factors. First, patients with personal or family histories of allergies are more susceptible to ADRs. Second,* Panax notoginseng* is the active component of Xueshuantong injection and can stimulate the immune system.

## 4. Discussion

The cases of anaphylactic shock caused by different drugs varied greatly. The abovementioned data and examples showed that doctors should pay more attention to the drug that frequently causes anaphylactic shock. The first reason that anaphylactic shock occurs is because TCM injection components are complex. They are composed of organic compounds, such as pigment, tannin, starch, protein, and other ingredients in colloidal form, which can stimulate the body's immune system and produce antibody-sensitized T lymphocytes to induce hypersensitivity [41]. Second, allergic shock correlates with the temperature and humidity of transportation and storage. In addition, TCM injection is unstable; if storage conditions do not meet certain requirements, the amounts of harmful components and unstable particles will increase, causing anaphylactic shock [42]. Third, the TCM injection with the cosolvent, stabilizer, and possible allergens may activate the H1 receptor of the skin tissue, causing histamine release and an increase in body reactivity [43]. The poor drug quality and insoluble particles are the main reasons underlying the occurrence of ADRs, so nurses must observe the medicine to ensure that it has no liquid crystals, turbidity, or sedimentation.

People of any age can suffer anaphylactic shock induced by TCM injection. The age distribution in studies was such that 5 cases of allergic shock appeared in patients under the age of 18, 108 cases were in patients aged 19 to 45, 106 cases were in patients aged 46 to 59, and 132 cases were in patients over 60 years old. ADRs occurred in patients of all ages, but the largest number of cases (350, 67.43%) was in patients above 45 years old. According to the data, Tanshinone IIA sodium sulfonate,* Erigeron* injection, Shengmai injection, and Xuesaitong injection (over 50% of those treated) were more likely to cause the elderly (>60 years old) to suffer anaphylactic shock. This may be because the elderly are susceptible to a variety of cardiovascular and cerebrovascular diseases with frequent usage of the injection. The more drug they were administered, the more likely they were to suffer an ADR. In addition, individual doses in elderly patients cause different degrees of decline in organ function. Compared to youth, the elderly have different sensitivities and drug tolerance and are thus prone to drug accumulation. However, to a surprising extent, there was a significant proportion of young people who suffered ADRs. This occurred for two main reasons. First, young people often have a good physique and are thus easily ignored by doctors. Second, youth take medications more frequently than other ages because of physical maturity. Otherwise, persons under the age of 18 are relatively fewer, mainly because this group accounted for a small proportion of total population taking TCM injection. Doctors should exercise precaution when treating minors as they are still in the growth stage, and their liver and kidney functions and some enzyme systems have not matured. In conclusion, before selecting TCM injections for treatment, doctors should ask about patient to detail their personal and family medical and drug allergy history. In addition, doctors should exercise caution when treating elderly people, children, and other special groups, such as patients with a history of drug allergies.

In general, there were no significant differences in gender with regard to anaphylactic shock; therefore, for the most part, doctors can equally consider the treatment of male and female patients with cardiovascular disease with TCM injection. However, some drugs did have gender-specific effects. For example, females were more prone than males to suffer anaphylactic shock after Shenmai injection and Xuesaitong injection, whereas males were more susceptible to anaphylactic shock after taking Breviscapine injection, Puerarin injection, and Xingnaojing injection.

Among the 350 people in the described studies, only 4 took TCM injection by IMI; the other patients were treated by IVI. At the same time, the ten death cases were received intravenously. Although IVI has a fast curative effect, treatment is more difficult when anaphylactic shock occurs. To reduce the incidence of anaphylactic shock, doctors should fully understand the drug indications, usage, dosage regimen, and route of drug administration. The drug should be administered orally, followed by IMI, with the final choice of drug administration by IVI or infusion. Use of Chinese herbal injections should be restricted to treatment of severe diseases or critical cases [1]. In addition, the patient should be prohibited from blindly taking a large dose and undergoing treatment for a long period of time, especially with IVI.

Together, the data showed that anaphylactic shock usually occurred between 3 sand 2.5 h, although there were some differences, depending upon a patient's fitness level, pathological state, genetic makeup, and drug sensitivity. In addition, TCM injections can more easily cause allergies when they are given as macromolecules; thus, this is the form that makes patients more susceptible to ADRs. Based on the abovementioned two reasons, the time of occurrence of anaphylactic shock differed between patients. Thus, the length of time that a patient should be monitored should be adjusted to suit that individual and in accordance with the type of drug administered. For example, patients who were administered Shenmai injection,* Salvia miltiorrhiza* injection, Dan Hong injection, compound Danshen injection, Mailuoning injection, Shengmai injection, or Xingnaojing injection suffered anaphylactic shock within 1 min. So, doctors need to pay close attention to patients who are treated with these drugs that cause immediate hypersensitivity. In addition, the appropriate emergency measures need to be immediately taken when serious ADRs occur. Patients who were administered Ciwujia injection, Breviscapine injection, compound Danshen injection, and Puerarin injection suffered anaphylactic shock after more than 2 h; thus, with these drugs, patients need to be observed for an extended period time. During the course of treatment, the drip rate should be strictly controlled, and the patient should be closely observed. Once an ADR occurs, treatment should be terminated, and the appropriate measures should be taken. I suggest that the patients should be observed more than two hours. The patients may leave after two hours, if they have no discomfort.

## 5. Conclusions

Based on the analyses of the aforementioned studies, we suggest the following. The first suggestion is for pharmaceutical enterprises. Chinese herbal injections should be approved by the SFDA according to the results of double-blind randomized controlled clinical trials [1]. Although TCM injections can cause allergic shock and other serious ADRs, instructions on how to use these drugs are rarely mentioned. In addition, some TCM injection instructions are not standardized, are poorly written, and lack information and warnings about possible clinical toxicities and side effects [44]. Therefore, pharmaceutical companies should equip TCM with detailed instructions, including possible ADRs and contraindications.

The second suggestion is for pharmaceutical supervisory and administrative departments. The ADRs caused by TCM injection are multifaceted, with diverse clinical manifestations. The production of TCM injection is extracted according to the theory of TCM and a precise process. The essence of TCM is Bianzhenglunzhi. According to Bian zheng lun zhi, the patient's health situation can be treated holistically. Holistic approaches may have their merits, especially in terms of promoting optimum health [3]. Therefore, the use of Chinese medicines should be guided by TCM theory and, in particular, should be according to the syndrome differentiation treatment principle. Doctors should not simply and blindly follow instructions, without allowing for differences between individual patients. They should pay attention to different diseases that have the same symptoms or one disease that has different symptoms, thereby achieving the goal of reasonably applying the principle to TCM injections [44]. This requires doctors to strictly master the application method of the drug and to build upon their knowledge of ADRs caused by TCM injections. In order to improve public safety, doctors should take measures to avoid the occurrence of ADRs to the extent possible.

The third suggestion is for the pharmacy department and pharmacists. Pharmacists must check prescriptions, especially those for TCM injections, to reduce the occurrence of repeated drug use, drug overuse, and irrational drug use. In addition, information on TCM injections, contraindications, and ADRs should be obtained and collected. Any observed ADRs should be reported to the hospital in a timely manner. Drug consultation services should be accessible that will provide accurate medical information to patients, thereby ensuring the reasonable, effective, and safe use of medication. In addition, clinical pharmacists should do ward rounds of the wards together with physicians for the purpose of strengthening the clinical supervisory activities and guiding the proper clinical use of the medications [45].

Fourth, it is important for patients to detail their personal and family history of allergies before being administered TCM injection. Patients who are allergic to medication should be closely observed after injection, and if any ADR occurs, doctors should immediately terminate drug administration [13].

All things considered, TCM injection has a unique role in medical practice, as it not only has the common advantages of injection, but also retains the characteristics of TCM. As a new dosage form of TCM, TCM injection has the advantages of being effective and accurately dosed compared with other TCM dosage forms, especially in treating the seriously ill. Importantly, TCM injection has overcome the inconvenience and difficulty in boiling process. The naysayers of this drug have brought great attention to the ADRs caused by TCM injection due to its complex composition, incomplete preparation process, and lack of rigorous evaluation of clinical efficacy. In order to maximize the effectiveness of this treatment and to reduce the associated ADRs, regulators are seeking to more strictly monitor and reevaluating these drugs and improve the relevant laws and regulations. Medical personnel should also cooperate with regulatory authorities to monitor and report ADRs and to take active preventative measures to reduce or avoid the occurrence of severe ADRs, thereby ensuring drug safety to the public [44].

## Supplementary Material

The Supplementary Material is the literature about data of 17 types of Traditional Chinese Medicine injections.

## Figures and Tables

**Table 1:** Patients who suffered anaphylactic shock after being treated with 17 types of traditional Chinese medicine injections for cardiovascular and cerebrovascular diseases.

Serial number	Chinese herbal injections	Constituents	Number of cases	Distributions of ages^*^	Sex	Mode of delivery	Onset of symptoms	Distributions of onset^*^	Timely rescue	Number of deaths
1	Shenmai injection	(1) Red ginseng (2) *Ophiopogon japonicus *	39	0/22/11/6	26 F, 13 M	36IVI^*^, 3IMI^*^	10 s–1 h	8/25/6/0/0	Yes	0

2	Ciwujia injection	Ciwujia stem and leaf extracts	33	0/11/14/8	15 F, 18 M	33IVI	3 min–2.5 h	1/15/13/3/1	Yes	0

3	Salvia miltiorrhiza injection	*Salvia miltiorrhiza* extract	9	0/5/2/2	3 F, 6 M	9IVI	1 min–30 min	2/4/3/0/0	Yes	0

4	Tanshinone IIA sodium sulfonate injection	*Salvia miltiorrhiza* extract; main components: Two terpene quinone compounds	5	0/0/1/4	3 F, 2 M	5IVI	2 min–30 min	0/1/4/0/0	Yes	0

5	Dan Hong injection	(1) *Salvia miltiorrhiza* extract (2) Safflower extract	4	0/2/0/2	3 F, 1 M	4IVI	1 min–40 min	0/3/1/0/0	Yes	0

6	Breviscapine injection	*Erigeron breviscapus* extract	11	0/2/4/5	3 F, 8 M	11IVI	3 min–2 h	0/2/6/3/0	Yes	0

7	Erigeron injection	*Erigeron breviscapus* extract: phenolic constituents Main component: wild Baicalin, total caffeic acid ester	6	0/0/0/6	4 F, 2 M	6IVI	10 min–1.5 h	0/1/4/1/0	Yes	0

8	Compound Danshen injection	(1) *Salvia miltiorrhiza* extract (2) Dalbergia extract	53	0/19/18/16	23 F, 30 M	53IVI	10 s–2 h	5/30/15/3/0	Yes	5 death, 1 fetal death

9	Puerarin injection	(1) Puerarin extract (2) *Propylene glycol*, water for injection	23	0/1/11/11	8 F, 15 M	23IVI	2 min–2 h	1/2/18/2/0	Yes	2

10	Safflower injection	Safflower extract Main components: safflower yellow pigment, safflower quinone, glycoside, carthamin, new carthamin	27	0/7/10/11	15 F, 12 M	27IVI	2 min–1 h	1/16/10/0/0	Yes	0

11	Mailuoning injection	Honeysuckle, *Achyranthes* root, *Dendrobium*, Xuan Can	64	1/19/15/29	30 F, 34 M	64IVI	10 s–50 min	11/40/13/0/0	Yes	2

12	Shengmai injection	Red ginseng, *Ophiopogon japonicus*, *Schisandra chinensis *	16	0/2/5/9	7 F, 9 M	16IVI	10 s–30 min	3/10/3/0/0	Yes	0

13	Shuxuetong injection	Leech and earthworm	6	0/1/3/2	4 F, 2 M	6IVI	5 min–1.5 h	0/3/2/1/0	Yes	0

14	Xingnaojing injection	Musk, borneol, turmeric, *Gardenia *	17	2/7/3/5	4 F, 13 M	17IVI	3 s–45 min	1/12/4/0/0	Yes	0

15	Xuebijing injection	Extracts: safflower, Radix Paeoniae Rubra, Rhizoma Chuanxiong, Radix *Salviae miltiorrhizae*, Radix *Angelicae sinensis* Main component: safflor yellow A	12	2/7/2/1	7 F, 5 M	12IVI	2 min–10 min	0/12/0/0/0	Yes	0

16	Xuesaitong injection	Total saponins of three-seven byproducts, main components: Ginsenoside Rb1, Ginsenoside Rg1, three-seven, Ginsenoside R1	21	0/3/5/13	13 F, 8 M	20IVI, 1IMI	2 min–1.5 h	1/11/8/1/0	Yes	0

17	Xueshuantong injection	Three-seven root extracts, main components: Ginsenoside Rg1, Ginsenoside Rb1	4	0/0/2/2	1 F, 3 M	4IVI	3 min–1 h	0/2/2/0/0	Yes	0

	Total		350	5/108/106/132	169 F, 181 M			34/189/112/14/1		

^*^Distributions of ages: number of children (≤18 years)/number of youth (19–45 years)/number of middle-aged (46–59 years)/number of aged cases (≥60 years).

^*^Distributions of onset: within 1 min/between 1 min to 10 mins/between 11 mins to 60 mins/between 61 mins to 120 mins/over 120 mins.

IVI: intravenous injection.

IMI: intramuscular injection.

The references of the cases are in the support material.
