# Supplementary material for: Analysis of Anaphylactic Shock Caused by 17 Types of Traditional Chinese Medicine Injections Used to Treat Cardiovascular and Cerebrovascular Diseases
Source: Biomed Res Int. 2015 Apr 27;2015:420607. doi: 10.1155/2015/420607 (PMC4426654; doi:10.1155/2015/420607)
Supplement: Supplementary file 1 — The Supplementary Material is the literature about data of 17 types of Traditional Chinese Medicine injections. [file 420607.f1.pdf]

Supporting material

References

Shenmai injection No.cases39

Chen YP, Ying Y. Analysis and nursing countermeasures of the adverse reaction of Shenmai injection. Strait Pharmaceutical Journal.2013;25(6):286-287

Xiao XP. One case of allergic reaction of Shenmai Injection. Seek Medical And Ask The Medicine.2012;10(9):84

Liu ZG, Yang S. Clinical analysis of 1 case of allergic reaction induced by Shenmai injection. Seek Medical And Ask The Medicine.2012;10(6):400-401

Liu LY. One case of anaphylactic shock induced by Shenmai injection. Nursing Practice And Research.2011;8(6):128

Wang L, Zhang JY, Gao JN. One case of immediate anaphylactic shock induced by Shenmai injection. Chinese Journal of Pharmacovigilance.2010;7(6):380

Zhang GX, Yang YJ, You DH. Clinical treatment and nursing of 2 cases of allergic reaction induced by Shenmai injection. Jilin Medical Journal.2010;31(3):413

Jiang XY, Ye YM, Liu JL. Analysis of allergic shock induced by Shenmai Injection.Chinese Journal of Ethnomedicine and Ethnopharmacy.2009;20:78

Hou WH, Feng CM, Cheng B, Chen QQ. Emergency of 1 cases of allergic shock induced by Shenmai injection. Journal of Qilu Nursing.2009;15(5):124

Cao Q, Shi JH. Analysis of one case with adverse reactions induced by intravenously infusion Shengmai injection. Chinese Nursing Research.2008;22(9B):2432

Li J. Rescue and nursing of 2 cases of allergic reaction induced by Shenmai Injection .Today Nurse.2008;9:88-89

Chen JW. One case of adverse reactions induced by Shenmai injection. Chinese Journal of Pharmacovigilance.2007;4(5):311

Tan XJ. Rescue of a case of immediate allergic reaction induced by Shenmai injection. Tianjin Journal of Nursing.2007;15(3):166-167

Zhang ZW, Fang L. One case of allergic shock induced by Shenmai injection. Medical Journal of National Defending Forces in Northwest China.2007;28(2):126

Wang AL, Wang YS. Intravenous Shenmai induced anaphylactic shock. China hydropower medicine.2006:4:236

Fei Y, Wang L. One case of adverse reactions induced by Shenmai injection. Journal of Southeast China National Defence Medical Science.2005;7(3):202

Wei LY. One case of allergic reactions induced by intravenous Shenmai injection. Jiangsu Pharmacertical and Clinical Research.2004;12:83

Chen Y. Nursing care of 1 case of severe allergic reaction caused by intravenous drip of Shenmai Injection. Modern Clinical Nursing.2004;3(5):59-62

Hu YL, Pan LQ, Li XJ. One case of allergic shock induced by Shenmai injection. Herald of Medicine.2004;23(9):694

Dai HD. One case of allergic shock induced by Shenmai injection. Chinese Journal of Epidemiology.2004;13(1):46

Yu XF. One case of allergic reactions induced by intravenous Shenmai injection. Chinese Journal of Modern Applied Pharmacy.2003;20(1):13

Wang HY, Lin Y, Cao JT. Two cases of adverse reactions induced by intravenous Shenmai injection. China Journal of Chinese Materia Medica.2002;27(7):558

Lu LR, Yang YF, Wang AR. Two cases of adverse reaction induced by Shenmai Injection.2002;1:62

He DY, Guo YF. One case of adverse reactions induced by Shenmai injection. Chinese Journal of Modern Applied Pharmacy.1997;14(3):55

Xiang DF. Three cases of adverse reactions induced by Shenmai injection. China Journal of Chinese Materia Medica.1996;21(6):378

Ren YS. Two cases of adverse reactions induced by Shenmai injection. Journal of Practical Traditional Chinese Medicine.1994;1:35

Yang L. Report of a case of allergic reaction induced by Shenmai Injection. Yunnan Journal of Traditional Chinese Medicine and Materia Medica.1996;17(2):58

Luo K. One case of allergic shock induced by Shenmai injection. Guangxi Journal of Traditional Chinese Medicin.1996;2:39

Zhang N. One case of allergic shock induced by Shenmai injection. Chinese Journal of Misdiagnostics.2011;11(4):1000

Lu JS. One case of allergic shock induced by Shenmai injection. Herald of Medicine.2003;22(1):56

Wu JP, Xu ZJ, Zhu LW. One case of allergic shock induced by Shenmai injection. Chinese Journal of Hospital Pharmacy.2000;20(1):64

Zhang ZX, Yuan B. One case of allergic shock induced by intravenous Shenmai injection. Liaoning Pharmacy and Clinical Remedies.2002;5(1):52

Wei YY. One case of serious allergic reaction induced by intravenous Shenmai injection. Journal of Youjiang Medical College For Nationalities.2001;2:293

Li L. One case of allergic reaction induced by Shenmai injection. Modern Journal of Integrated Traditional Chinese and Western Medicine.2001;10(13):1274

Li CY, Wang JL. One case of allergic reaction induced by intravenous Shenmai injection. Nanjing Army Medical.2002;4(6):47

Li L. Report of one case of allergic reaction induced by Shenmai injection. Journal of Sichuan of Traditional Chinese Medicine.1991;5:12-13

Liu JF, Wu LB. One case of delayed allergic shock induced by Shenmai injection. Chinese Remedies&Clinics.2004;(10):807

Ciwujia injection No.cases33

Deng WD. Analysis of 2 cases of Allergic reaction induced by Ciwujia injection. Journal of Chinese Practical Diagnosis and Therapy. 2007;21(3):218-219

Yan H, Luo HP. Allergic reaction induced by Ciwujia injection. China Practical Medicine.2007; 2(22):11

Ding BY, Zhao HY, Dai ZQ. One case of allergic reaction induced by intravenous Ciwujia injection. Chinese Journal of Drug Application and Monitoring.2007;1:62

Luo Y. Nursing of allergical respiratory arrest induced by Intravenous Acanthopanax senticosus injection. Guangdong Medical Journal.2006;27(12):1934

Guan HW, Zhang W. Clinical observation and nursing of allergic reaction induced by Ciwujia injection. Journal of Practical Traditional Chinese Internal Medicine.2006;3

Mao YJ, Liang JY. Three cases of allergic shock induced by Ciwujia injection. Chinese Journal of Modern Applied Pharmacy.2006; 23(2):174

Xu M. One case of allergic shock induced by Ciwujia injection. Nanfang Journal of Nursing.2005;12(10):23

Li YH, Luo HY. One case of allergic shock induced by Ciwujia injection.Medicine World.2005;17(7):691

Jiang CP, Wu BH. Two cases of allergic shock induced by Ciwujia injection. Journal Of North Sichuan Medical College.2002;17(2):115-116

Liu WZ, Ren L. One case of allergic shock induced by Ciwujia injection. Modern Journal of Integrated Traditional Chinese and Western Medicine.2002; 11(7):647

Zhou Y, Wang GG. One case of serious allergic shock induced by refined Ciwujia injection. Tibetan Journal of Medicine.2001;22(2):72

Xie SG. Two cases of allergic shock induced by Ciwujia injection. Strait Pharmaceut ical Journal.2000;12(4):88-89

Du LM, Wang BX, Li XZ. One case of allergic shock induced by Ciwujia injection. Linyi Medical College Writing.1997;19:250

Zhu BH, Hu J. Three cases of allergic shock induced by intravenous Ciwujia injection. Chinese Journal Of Rural Doctor.1998;10:45-46

Chi XH. .A Case of Acanthopanax Root Allergic Reaction Induced Death. Chinese Nursing Research.2003;17(1B):67

Xu JH, Zhang QY. Three cases of adverse reaction induced by Ciwujia injection. Xinjiang Journal of Traditional Chinese Medicine.1997;15(2):50

Zhang YK. One case of allergic shock induced by Ciwujia injection. Chinese Journal of Hospital Pharmacy.1998;18(12):574

Yang JZ, Zhang RP, Chen YL. One case of allergic shock induced by Ciwujia injection. Chinese Journal of Hospital Pharmacy.1998;18(12):574

Wang YD, Li HF, Chen SX. One case of allergic shock and visual vertigo induced by Ciwujia injection. Chinese Journal of Hospital Pharmacy.1998;18(12):574

You F, Bai Y M. One case of serious allergic reaction induced by Ciwujia injection. Chinese Journal of Hospital Pharmacy.1998;18(7):329

Si FB, Hu SF, Zhang C. Three cases of allergic shock induced by intravenous Ciwujia injection. Chinese Journal of New Drugs.1996;5(5):369-370

Li KS. One case of serious allergic reaction induced by intravenous Ciwujia injection. China Journal of Chinese Materia Medica.1998;23(7):435

Wang XD, Ren BY, Zhang L. Report of one case of serious allergic reaction induced by Ciwujia injection. Harbin Medical Journal.1997;17(3):48

Kang JH, Fu D, Guo CX. One case of allergic shock induced by Ciwujia injection. The Chinese Journal of Clinical Pharmacology.1997;4:232

Lin ZP, Zhao YX. One case of serious allergic shock induced by intravenous Ciwujia injection. Chinese Journal of Pharmacoepidemiology.1999;8(2):110

Salvia miltiorrhiza injection No.cases9

Zhang L, Chen HJ, Wang XW. One case of Salvia miltiorrhiza injection intravenous drip too fast induced allergic reaction. Nursing Practice and Research.2008;21

Jiang HQ, Chen SY. One case of high concentration Salvia miltiorrhiza injection induced hypovolemic shock. Strait Pharmaceutical Journal.2000;12(4):102

Jiang CG. One case of allergic reaction induced by Salvia miltiorrhiza injection. China Journal of Chinese Materia Medica.1994;19(8):503

Zhu ZM, Zhou FL. One case of allergic shock induced by Salvia miltiorrhiza injection with Dextran 40 injection. Herald of Medicine.1995;14(5):231

Li QY. One case of allergic reaction induced by Salvia miltiorrhiza injection. Journal of Traditional Chinese Ophthalmology.1999;9(2):110

Ding F, Xue J. Two cases of allergic reaction induced by Salvia miltiorrhiza. Modern Journal of Integrated Traditional Chinese and Western Medicine.2000;9(9):852

Chen SH. One case of allergic shock induced by Salvia miltiorrhiza injection. Journal of Occupational Health and Damage.2006;21(4):313

Zhu YX, Liu GX, Lv XQ, Chen XL. Two cases of allergic shock induced by Salvia miltiorrhiza with Dextran 40. Journal of Clinical Dermatology.1998; 27(4):220

Tanshinone IIAsodium sulfonate injection No.cases5

Zhu JS, Zhou JM. Four cases of adverse reaction induced by Tanshinone II a sodium sulfonate injection. Chinese Journal of Drug Abuse Prevention and Treatment.2012;18(1):60-61

Long XC. One case of Tanshinone II a sodium sulfonate injection induced hypovolemic shock. Chinese Journal of Misdiagnostics.2011; 11(15):3769

Li M. Analysis of one case of adverse reaction induced by Tanshinone II a sodium sulfonate injection.Journal of Pharmaceutical Practice.2010;28(6):473-474

Chen XY. One case of adverse reaction induced by intravenous Tanshinone II a sodium sulfonate injection Chinese Journal of Misdiagnostics.2010;10(25):6054

Fei Y, Wang L, Huang HL. Two cases of adverse reaction induced by Tanshinone II a sodium sulfonate injection. Strait Pharmaceutical Journal.2005;17(2):154-155

Dan Hong injection No.cases4

Bao ZY, Qiu S. Allergic reaction induced by Danhong injection. Adverse Drug Reactions Journal.2009;11(6):447

Wang HB, Li JC, Jin SP. The adverse reaction of Danhong injection. Chinese Journal of Misdiagnostics.2009; 9(28):7044-7045

Yuan DM, Zhao SK. One case of Danhong injection induced serious adverse reaction. Chinese Pharmaceutical Affairs.2009;23(10):1047-1048

Zheng SQ, Li YF. One case of allergic reaction induced by intravenous Danhong injection. Chinese Journal of Misdiagnostics.2007; 7(13):2942

Breviscapine injection **No.cases11**

Yue YM. One case of allergic reaction induced by Breviscapine Injection. Chinese Journal of Clinical Rational Drug Use.2012;5(12A):8

Liu YP, Bai SQ. Analysis of twelve cases of adverse reaction induced by Breviscapine Injection in our hospital. Nei Mongol Journal of Traditional Chinese Medicine.2012;4:49-50

Zhu LP, Liu CY. One case of adverse reaction induced by Breviscapine Injection. Chinese Journal of Pharmacoepidemiology.2007; 16(4):247-248

Liu L, Li AY, Lu XJ. Two cases of adverse reaction induced by Breviscapine Injection. China's Naturopathy.2005;13(7):52-53

Zhao H, Li CJ. The adverse reaction of Breviscapine Injection. Chinese Journal of Misdiagnostics.2004;4(7):1135-1136

Shi YP. Two cases of adverse reaction induced by Breviscapine Injection.Chinese Traditional Patent Medicine.2002;24(9):730

Li RQ, Lin S. Three cases of adverse reaction induced by Breviscapine powder-Injection. Chinese Journal of Hospital Pharmacy.1997;17(7):330

Erigeron injection No.cases6

He NS, Zhao YJ. Three cases of allergic reaction induced by Erigeron Injection.Chinese Journal of Information on TCM.2005;12(1):82-83

Lei L, Chen Y, Zhao YJ. Three cases of allergic reaction induced by Erigeron Injection. Chinese Pharmaceutical Affairs.2004;18(11):702-703

compound Danshen injection No.cases53

Hong YF. The adverse reaction and preventive measure of Compound Danshen injection. Chinese And Foreign Medical Research.2011;9(24):169-170

Ma JQ, Yang WH, Shi XJ. Analysis of one case of allergic shock induced by Compound Danshen injection. Chronic Pathematology Journal.2009;11(4):83-84

Gao YC, Su LL, Zhao ZG. Report of one case of allergic reaction induced by Compound Danshen injection. Chinese Pharmaceutical Journal.2007; 42(18):1439

Liu HJ, Liu YN. Analysis of sixteen cases of adverse reaction induced by Compound Danshen injection. Xinjiang Journal of Traditional Chinese Medicine.2006;24(3):34-35

Wang SH, Tian XJ. One case of allergic shock induced by Compound Danshen injection. Chinese Journal of Rural Medicine and Pharmacy.2005;12(9):9

Tian XK. The adverse reaction of Compound Danshen injection. Hebei Journal of Traditional Chinese Medicine.2004; 26(10):786

De QZG. Two cases of allergic reaction induced by Compound Danshen iInjection . Chinese Journal of Modern Applied Pharmacy.2003; 20(1):77

Li ZZ. Two cases of allergic shock induced by Compound Danshen injection . Chinese Journal of Rural Medicine and Pharmacy.2002;9(11):35-36

Niu Y. One case of allergic reaction induced by intravenous Compound Danshen injection with glucose injection. Practical Journal of Medicine & Pharmacy.2002;19(10):733

Huang RJ, Huang L. Allergic shock induced by intravenous Compound Danshen injection . Adverse Drug Reactions Journal.2002;4:263

Li Y. One case of allergic shock induced by intravenous Compound Danshen injection. Modern Diagnosis & Treatment.2001; 12(6):324

Zhang YJ, Zhang Q, Liu Y. Report of three cases of allergic reaction due to Compound Danshen injection. Aerospace Medicine.2001;12(4):245-246

Wang AR, Wang CY. One case of allergic shock due to intravenous Compound Danshen injection. Lishizhen Medicine And Materia Medic Research.2001;12(3):278

Zhou ZL, Liu ZX. One case of allergic shock due to Compound Danshen injection. Journal of Hubei Institute for Nationalities(Medical Edition) .2001;18(1):45

Rong WN, Rong W.H. Two cases of allergic reaction due to Compound Danshen injection. Herald of Medicine.2000;19(3):302-303

Wan MCM. Two cases of allergic shock due to Compound Danshen injection. Qinghai Medical Journal.2000;30(6):38

Zhu XF, Huang WL. One case of allergic reaction due to Compound Danshen.Strait Pharmaceutical Journal.1997;9(1):145

Zhong SC. One case of allergic shock due to Compound Danshen injection. China Journal of Chinese Materia Medical.1994;9(10):631

Yang BZ. One case of allergic shock due to Compound Danshen injection. Tianjin Journal of Traditional Chinese Medicine.1992;5:48

Ming ZX. One case of sudden death due to Compound Danshen injection. Herald of Medicine.1997;16(6):299

Chen YS, Wang GR. One case of allergic shock due to Compound Danshen injection. Journal of China-Japan Friendship Hospital.1996;10:166

Cao ZT, Wang ZQ, Zhuo Y. One case of cardiac arrest caused by low molecular dextran with Compound Danshen .Anhui Medical Journal.1995;16(1):62

Wang H, Zhong B. One case of shock to death caused by low molecular dextran with Compound Danshen .Chinese Journal of Hospital Pharmacy.1997;17(9):429

Zhang GQ. Two cases of allergic reaction due to Compound Danshen injection. Chinese Journal of Rural Medicine and Pharmacy.2004;11(12):45

Zhu YQ. One case of allergic reaction due to Compound Danshen injection. Journal of Baotou Medicine.2000;24(1):45

Bao CF. One case of allergic shock due to Compound Danshen injection. Modern Journal of Integrated Traditional Chinese and Western Medicine.2000;9(13):1282-1283

Huang ZY, Jing Y. One case of allergic shock due to intravenous Compound Danshen injection. Journal of Internal Intensive Medicine.2001;7(1):11

Gao H, Yang JY. Two cases of allergic shock due to Compound Danshen injection. Chinese Journal of Misdiagnostics.2004;4(10):1755

Wang AS. One case of allergic shock due to intravenous Compound Danshen with low molecular dextran. Journal of Qiqihar Medical College.2001;3

Mai QY. One case of allergic shock to death due to intravenous Compound Danshen injection. Chinese Journal of Difficult and Complicated Cases .2005;4(1):61

Liang GZ, Xia H. Three cases of serious adverse reaction caused by Dextran 40 mixed with Compound Danshen injection. Chinese Journal of Hospital Pharmacy.1995;15(9):425-426

Liu W, Yan MY. One case of allergic shock due to Compound Danshen injection. Clinical Journal of Medical Officer.2006; 34(4):423

Lu JJ, Chen YH. Two cases of allergic shock due to Compound Danshen injection. Clinical Misdiagnosis& Mistherapy.2006;19(7):90

Li YL, Zhang YP, Zeng P. Analysis of pregnant women’s intravenous drip of low molecular dextran and Compound Danshen injection induced allergic shock and

intrauterine fetal death. Chinese Journal of Integrated Traditional and Western Medicine.2003;23(1):21

Shi XL, Zhang Y. One case of adverse reaction caused by low molecular dextran mixed with Compound Danshen injection. Northwest Pharmaceutical Journal.2007;22(5):276

Li J, Jing T. One case of allergic shock due to Compound Danshen injection. Jilin Journal of Traditional Chinese Medicine.2005;25(2):25

Liu LY. One case of allergic shock due to Compound Danshen injection. Applied Journal of General Practice.2007;5(6):546

Wu JL. One case of allergic shock due to Compound Danshen injection.Journal of Dermatology and Venereology.1997;19(3):67

Zhou HJ, Meng XZ. Allergic shock due to Compound Danshen injection. Heilongjiang Journal of Traditional Chinese Medicine.2002;3:47

Dai ZM, Huang SL. Allergic shock due to low molecular dextran mixed with Compound Danshen injection. Strait Pharmaceutical Journal.1995;7(2):45

Cai LJ, Zeng QL, Liu XY. Two cases of allergic reaction caused by intravenous Compound Danshen injection mixed with low molecular dextran.Strait Pharmaceutical Journal.1995;7(3):38-39

Wei KL, Liu ZG, Chen XY. Allergic shock due to Compound Danshen injection. Journal Of Binzhou Medical College.2001;24(3):228

Puerarin injection No.cases23

Sun YW. Adverse reaction of Puerarin injection. Chinese Community Doctors 6,19-20

Li,G.,Lou,X.,2010. Analysis of 8 cases of adverse reaction due to Puerarin injection. Chinese Community Doctors.2011;8:157

Wang WW, Yu ZL, Wang MH, Wang QS. One case of allergic shock caused by Puerarin injection. Chinese Journal of Hospital Pharmacy.2009;29(9):780

Wang HL, Zhang Y. Adverse reaction of Puerarin. Chinese Journal of Misdiagnostics.2007;7(8):1915

Chen JP. Report of a case of chills and chest tightness caused by Puerarin and Glucose Injection. Qinghai Medical Journal.2006;7(8):1915

Liu G.Z, Dong F. One case of allergic reaction caused by Puerarin injection. Herald of Medicine.2005;24(1):6

Zheng YH. Five cases of serious adverse reaction due to Puerarin. Modern Practical Medicine.2004;16(1):38

Xu F, He KN. Rare serious adverse reaction due to Puerarin. Chinese Journal of Integrated Traditional and Western Medicine.2003;23(5):362

Zheng YN. Four cases of allergic reaction caused by Puerarin injection. Journal Of Chengde Medical College.2002;19(3):251

Xia YH. One case of allergic reaction caused by Puerarin injection. Chinese Journal of Hospital Pharmacy.1999;19(2):128

Qiu SZ. Report of 1 case of allergic shock caused by Puerarin injection. China Journal of Modern Medicine.2000;10:54

Wang Y, Chen W, Wang YZ. Three cases of allergic shock caused by Puerarin injection. Chinese Pharmaceutical Journal.2000;35(5):353

Ye YP, Liu YX, Chen MH. Report of 4 cases of allergic reaction caused by Puerarin injection. Clinical Focus.2001;16(14):660

Zhang ZL, Yin T. Three causes of deaths caused by Puerarin injection. Adverse Drug Reactions Journal.2004;1:41

Zhu JZ. Allergic shock due to Puerarin injection. Adverse Drug Reactions Journal.2004;1:42-43

Wang XX, Yu YF, Liu AL. One case of allergic shock caused by Puerarin injection. Chinese Journal of Drug Application and Monitoring.2006;1:64

You GS. Two cases of allergic reaction caused by Puerarin injection. Chinese Community Doctors.2007;24:190

Li SE, He SP. Five cases of allergic reaction caused by Puerarin injection. Chinese Journal of Pharmacoepidemiology.1997;6(4):251

Safflower injection No.cases27

Lu YX, Ye QY. Nursing experience of one case of allergic shock due to Safflower injection. Chinese And Foreign Medical Research.2013;11(23):122

Tao TG.. Report of one case of serious allergic reaction due to Safflower injection. Clinical Rational Drug Use.2012;5(5C):161

Tang LJ, Mo Y. Analysis of adverse reaction due to Safflower injection. Clinical Rational Drug Use.2011;4(6C):70-71

Yu WM, Xu FM, Xu XF. Report of one case of allergic shock due to Safflower injection. Nei Mongol Journal of Traditional Chinese Medicine.2011;5:123

Han JY, Gao KS. Two cases of adverse reaction due to Safflower injection. Health Vocational Education.2009;27(5):155

Yang XZ, Bai TL. Report of one case of allergic shock due to Safflower injection. Qilu Pharmaceutical Affairs.2008;27(12):758

Xu Q, Liao Q, Zeng YL. One case of serious allergic reaction due to Safflower injection. Chinese Journal of Hospital Pharmacy.2008;28(21):1894

Ma MC, Ma LP. One case of allergic shock due to Safflower injection. Medical Journal of National Defending Forces in North China.2006;18(4):303

Song FJ, Hu TX. One case of allergic shock due to Safflower injection. Nursing Practice and Research.2006;4

Liu XH. Clinical report of allergic shock caused by Safflower injection. The Journal of Traditional Chinese Orthopedics and Traumatology.2005;17(9):44

Li GY. Serious allergic reaction due to Safflower injection. Adverse Drug Reactions Journal.2003;4:270

Ji WX. One case of allergic shock due to Safflower injection. Pharmaceutical Care and Research.2003;3(1):3

Gong CY, Gao Q, Yang H. One case of allergic reaction due to Safflower injection. Herald of Medicine.2000;19(6):609

Han YB. One case of allergic shock due to Safflower injection. Journal of Huaihai Medicine.2003;21(6):504

Wu YX, Han C, Guo P. One case of acute allergic reaction due to Safflower injection. Shanxi Medical Journal.2007;36(10):950

Li ZX. One case of allergic reaction due to Safflower injection. Chinese Journal of Misdiagnostics.2007;7(13):3172

Hu B, Tan ZP, Wang HC. One case of allergic respiratory difficulties caused by Honghua Injection. Chinese Journal of Pharmacoepidemiology.2006;15(5):309-310

Pan Y. One case of allergic shock due to Safflower injection. Herald of Medicine.2005;24(7):650

Wang CR, Bi JH. One case of allergic shock due to Safflower injection. Chinese Journal of Drug Application and Monitoring.2004;3:38

Guo LH, Feng XM, Zhang Q. Rescue experience of allergic shock caused by Safflower injection. Journal of Yunyang Medical College.2007;26(6):378

Du GA, Wang RF, Wang GS, Li YJ. Honghua injection-induced serious allergic reaction. Adverse Drug Reactions Journal.2004;6:373

Li JR, ,Huang YR. Two cases of allergic reaction due to Safflower injection. Xinjiang Journal of Traditional Chinese Medicine.2007;25(2):16

Yu H. One case of allergic shock due to Safflower injection. Chinese Journal Of Clinical Pharmacy.2004;13(2):115

Fan XJ, Bai SM, Xue L. One case of allergic shock due to Safflower injection. Shanxi Medical Journal.2007;36(1):6

Du J, Si DY. One case of serious allergic shock due to Safflower injection. Shenyang Army Medical.2005;18(4):253

Mailuoning injection No.cases64

Zhang NY. One case of adverse reaction caused by Mailuoning injection. Shenyang Army Medical.2011;24(3):155

Guan YJ, Li SM, Li JC. Adverse reaction of Mailuoning injection. Disease monitor & control.2011;5(4):239-240

Han JX, Yan F. Report of one case of adverse reaction due to Mailuoning injection.China Practical Medicine.2011;6(11):209-210

Zhang SG, Hou T, Yan RZ. One case of allergic shock due to Mailuoning injection. Qilu Pharmaceutical Affairs.2009;28(6):380

Zhong XY, Qiu P, Liu GZ. One case of adverse reaction due to Mailuoning injection. Journal of Changchun University of Traditional Chinese Medicine.2008;24(3):307

Wu QQ. Nursing experience of one case of allergic shock due to Mailuoning injection. China Practical Medicine.2008;3(8):134

Han YZ, Zhang WS. Three cases with dyspnea caused by Mailuoning Injection Aerospace Medicine.2007;18(2):89

Liu GS. Analysis of one case of allergic reaction due to Mailuoning. Journal Of Practical Traditional Chinese Internal.2004;18(3):196-197

Chen W, Li XP. One case of allergic shock due to Mailuoning injection. Chinese Journal of Dermatovenereology of Integrated Traditional and Western Medicine.2003;2(3):148

Chen XH, Zhang NN. Two cases of drug reaction due to Mailuoning injection. Herald of Medicine.2003;22(8):539

Zhou J. One case of allergic shock due to Mailuoning injection. Herald of Medicine.2003;22(6):424

Wang SY, Ma AB. One case of allergic reaction due to Mailuoning injection. Modern Journal of Integrated Traditional Chinese and Western Medicine.2002;11(17):1731

Wang LP, Liu BL. One case of serious adverse reaction due to Mailuoning injection. Heilongjiang Medicine and Pharmacy.2002;25(3):110

Li MZ, Sun KH. Five cases of adverse reaction due to Mailuoning injection. Adverse Drug Reactions Journal.2002;3:200-201

Li N, Wei YP. Three cases of adverse reaction due to Mailuoning injection. Journal of Xinxiang Medical College.2001;18(6):401

Zhang LM. Two cases of adverse reaction due to Mailuoning injection. Lishizhen Medicine And Materia Medica Reserch.2001;12(9):858

Luan YH, Huang WQ. One case of allergic shock caused by Mailuoning injection. Herald of Medicine.2000;19(4):372

Zhang C, Yan L, Yang SM. One case of allergic reaction caused by Mailuoning injection. Journal of Traditional Chinese Ophthalmology.1999;2:109

Ge JP, Zhang YP. Report of 3 cases allergic reaction caused by Mailuoning injection. Xinjiang Journal of Traditional Chinese Medicine.1995;4:45

Yan CL, Yan YW. One case of allergic shock caused by Mailuoning injection. Hebei Medical Journal.1997;19(2):108

Pang GF, Li BE. One case of allergic reaction caused by Mailuoning injection. Traditional Chinese Medicine Research.1995;3:35

Liu ZQ. Three cases of allergic reaction caused by Mailuoning injection. Chinese Journal of Hospital Pharmacy.1996;16(4):187

Liu AX, Qiao GD, Cheng XJ, Song SZ. Two cases of allergic shock caused by Mailuoning injection. Northwest Pharmaceutical Journal.2000;15(6):287

Yu ZG, Wang Y. One case of allergic shock caused by Mailuoning injection. Chinese Journal of Hospital Pharmacy.1997;17(4):188

Lin X. One case of allergic reaction caused by Mailuoning injection. Xinjiang Journal of Traditional Chinese Medicine.1994;1:15-16

Yang M. Two cases of serious allergic reaction caused by Mailuoning injection. China Journal of Chinese Materia Medica.2000;25(12):745

Liu PY. One case of allergic shock caused by Mailuoning injection. Inner Mongolia Medical Journal.2003;35(1):94

Yue JF, Wang HT. One case of allergic shock caused by Mailuoning injection. China's Naturopathy.2002;10(11):41

He CX. One case of allergic reaction caused by Mailuoning injection. Journal Of Jinggangshan Medical College.2001;8(4):100

Zhou FL. One case of allergic shock caused by Mailuoning injection. Qianwei Journal of Medicine & Pharmacy.2000;17(5):318

Chen C. One case of allergic shock caused by Mailuoning injection. Ningxia Medical Journal.2001;23(3):141

Zhang XH, Zhang PY, Liu W, Fan QY. One case of allergic shock caused by Mailuoning injection. Chinese Journal of Integrated Traditional and Western Medicine.2001;21(4):311

Liu SQ. Report of one case of allergic shock caused by Mailuoning injection. Journal of Hebei Traditional Chinese Medicine and Pharmacology.1999;14(1):26

Fu MH. One case of allergic shock caused by Mailuoning injection. Journal of Huaihai Medicine.1998;16(3):61

Li YG. Report of one case of allergic shock caused by Mailuoning injection. Journal Of Snake.1997;9(2):17

Qing XR. Two cases of allergic shock caused by Mailuoning injection. Xinjiang Journal of Traditional Chinese Medicine.1997;15(1):37-38

Yu H, Zhao QY. Two cases of serious allergic reaction caused by Mailuoning injection. Journal Of Baotou Medical College.2000;18:156

Li WP, Li H, Li SC. Two cases of allergic reaction caused by Mailuoning injection. Journal of Hebei Medical College for Continuing Education.2002;3:25

Han JL, Jiang Y, Sun CH. Emergency nursing of 2 cases of anaphylaxis induced by Mailuoning injection. Heilongjiang Medicine and Pharmacy.2001;24(3):90

Sui GK. One case of allergic reaction caused by Mailuoning injection. New Drug and Clinical Remedies.1993;12(2):103

Cheng ZR. Report of one case of allergic reaction caused by Mailuoning injection. Journal Of Practical Traditional Chinese Medicine.2002;18(3):51

Wang SY. Report of two cases of chest tightness caused by Mailuoning injection. Journal Of Jinggangshan Medical College.2001; 8(6):135

Wei W, Cao JQ. One case of Dyspnea caused by Mailuoning injection.Chinese Pharmaceutical Affairs.2000;14(3):201

Fu WN, Huang YM. Report of two cases of allergic reaction caused by Mailuoning injection. Chinese Journal of Convalescent Medicine.2001;10(6):7

Zhao CX. Report of one case of allergic reaction caused by Mailuoning injection. Nursing Journal of Chinese People's Liberation Army.2000;17(3):59

Tang J, Zhao SK. Report of one case of allergic reaction caused by Mailuoning injection. Journal Of Hebei Traditional Chinese Medicine And Pharmocology.1996;11(2):47

Li X. One case of allergic shock occurring after taking Mailuoning injection. Chinese Journal of Pharmacoepidemiology.1994;3(4):230-231

Wang SZ, Hu YY. One case of allergic reaction caused by Mailuoning injection. Xinjiang Journal of Traditional Chinese Medicine.1996;1:18

Zhao JR, Zhang Y. One case of allergic reaction caused by Mailuoning injection. People's Military Surgeon.2006;49(9):556

Shengmai injection No.cases16

Zeng MZ, Wang YH. One case of acute allergic reaction caused by Shengmai injection. China Pharmaceuticals.2013;22(13):84

Jin YZ, Da CL.. Five cases of adverse reaction caused by Shengmai injection. Chinese Journal of Drug Application and Monitoring.2009;6(3):192-193

Yan KX, Xu XY, Fu HZ. Nursing experience of one case of allergic reaction caused by Shengmai injection. Journal of Qilu Nursing.2008;14(1):85-86

Wei XJ, Zhao SQ. One case of allergic reaction caused by Shengmai injection. Herald of Medicine.2005;24(12):1151

Deng YH, Su LM. Two cases of adverse reaction caused by Shengmai injection. Shandong Medical Journal.2005;45(32):61

Zhang YP, A TK. Report of 3 cases of allergic reaction caused by Shengmai injection. Xinjiang Journal of Traditional Chinese Medicine.1998;16(2):19

Chen YQ, He YP. One case of serious allergic reaction caused by Shengmai injection. Heilongjiang Journal of nursing.2000;6(7):61

Gu CG.. Report of 1 case of allergic reaction caused by Shengmai injection. Chinese Journal of Modern Applied Pharmacy.1999;16(6):64

Lian M, Xing B, Cui CM. One case of allergic shock caused by Shengmai injection. Chinese Journal of Misdiagnostics.2007;7(3):662

Ye SL. One case of allergic shock caused by Shengmai injection. Chinese Journal of Critical Care Medicine.2001;21(12):737

Lin HR, Zhong JM. One case of allergic shock caused by Shengmai injection. Act Academia Medicine Weifang.2001;23(1):10

Te LHZ, Jia ZL. Shengmai injection induced allergic shock. Anaphylactic shock in association with Shengmai injection. Adverse Drug Reactions Journal.2007;9(2):80

Li CY. Anaphylactic shock due to shengmai injection. Adverse Drug Reactions Journal.2005;4:307

Yang XY. One case of allergic shock caused by Shengmai injection. Clinical Journal of Medical Officer.2009;37(5):780

Shuxuetong injection No.cases6

Li R, Wang C. Analysis of one case of allergic shock caused by Shuxuetong injection. Clinical Rational Drug Use.2011;4(9C):139-140

Liao CH. First aid nursing experience of allergic reaction induced by Shuxuetong injection. Journal of Clinical and Experimental Medicine.2007;6(11):191

Zhu XP. One case of allergic shock caused by Shuxuetong injection. Modern Journal of Integrated Traditional Chinese and Western Medicine.2007;16(2):238

Sun GF. One case of allergic reaction caused by Shuxuetong injection. China Journal of Chinese Materia Medica.2005;30(21):1697

Fei BH. Two cases of allergic reaction caused by Shuxuetong injection. China Pharmaceuticals.2006;15(9):63

Xingnaojing Injection No.cases17

He ZF, YangK, LiuN, Yan BL. One case of allergic reaction caused by Xingnaojing injection. Chinese Journal of Hospital Pharmacy.2013;33(2):169

Kong FF, Zhang SL, Guo LJ, Tan XQ. One case of allergic reaction caused by Xingnaojing injection. China Pharmaceuticals.2011;20(23):90

Wang X. One case of allergic reaction caused by Xingnaojing injection. Sichuan Medical Journal.2010;31(1):129

Dong CX, Wang ZY. Two cases of adverse reaction caused by Xingnaojing injection. Qilu Pharmaceutical Affairs.2009;28(12):757

Lai SC, Lin JH, Luo TH, Xiu LX. One case of allergic reaction caused by Xingnaojing injection. Strait Pharmaceutical Journal.2005 ;17(4) :182

Chen QH, Yu RM, Du JF. One case of adverse reaction caused by Xingnaojing injection. Hebei Medicine.2003;9(7):671

Tao L, Feng M. One case of allergic shock caused by Xingnaojing injection. Chinese Journal Of Clinical Pharmacy.2008;17(4):254

Yu HQ, Ma XH. One case of allergic shock caused by Xingnaojing injection. China Pharmacist.2008;11(12):1535-1536

Zhao B, Wang Q. Two cases of serious allergic reaction caused by Xingnaojing injection. Nursing Journal of Chinese People's Liberation Army.2002;19(3):32

Yin JZ. One case of allergic reaction caused by Xingnaojing injection. China Pharmacist2005;8(4):350

Sun XH, Li P. One case of allergic reaction caused by Xingnaojing injection. Heilongjiang Nursing Journal.1998;4(4):43

Feng ZQ, Chen BZ. One child of allergic reaction caused by Xingnaojing injection. Strait Pharmaceutical Journal.2001;13(1):90

Xue LL, You JC. Two cases of allergic reaction caused by Xingnaojing injection for treatment of traumatic brain injury. Heilongjiang Nursing Journal.2000;

6(1):32

Cui DZ. One case of allergic reaction caused by Xingnaojing injection. Shandong Medical Journal.1995;35(11):58

Xuebijing injection No.cases12

Huang HQ, Xu WT, Xu Y. One case of anaphylactic reactions caused by intravenous injection of Xuebijing injection. Symposium of Hainan Province pharmaceutical.2010;

Xu JT, Cai ZH. One case of immediate allergic shock caused by Xuebijing injection. Chinese Journal of Modern Drug Application.2009;3(2):126-127

Yang P, Yuan HN, Ma XH. Rescue and nursing of one case of allergic reaction caused by Xuebijing injection. Chinese Journal of Misdiagnostics.2008;8(23):5714

Wang P, Feng B. One case of allergic reaction caused by Xuebijing injection. Chinese Journal of Pharmacoepidemiology.2007;16(1):24

Hu XN. Two cases of allergic reaction caused by intravenous injection of Xuebijing injection. Clinical Journal of Medical Officer.2011;39(3):465

Wang N, Yue HX. Two cases of allergic reaction caused by Xuebijing injection. Medical Journal of National Defending Forces in Southwest China.2010;20(9):996

Zhao LN, Jiao LQ, Yue HX, Li X. One case of allergic reaction caused by Xuebijing injection. Journal of Emergency in Traditional Chinese Medicine.2008;17(10):1344

Sun HM, Wang GJ, Zhu QH. One case of allergic shock caused by Xuebijing injection. Chinese Pediatrics of Integrated Traditional and Western Medicine.2009;1(3):295

Zhang J. One case of allergic reaction caused by Xuebijing injection. Infection Inflammation Repair.2007;8(3):180

Ma J, Li L, Qian XQ. One case of immediate allergic reaction caused by Xuebijing injection. Chinese Journal of Clinical Healthcare.2010;13(4):429

Xuesaitong injection No.cases21

Gou CX, Ding CL. Analysis of 2 cases of adverse reaction due to Xuesaitong injection. Qilu Pharmaceutical Affairs.2012;31(11):678

Huang JZ. One case of allergic shock caused by intravenous injection of Xuesaitong injection after drinking. Chinese Journal of Rural Medicine and Pharmacy.2011;18(10):47

Wang Y, Wang M. Four cases of adverse reaction due to Xuesaitong injection.Chinese Journal of Pharmacovigilance.2007;4(4):248-249

Zhang YH, Dou WS, Yang YF, Zhan ML, XinRF. Three cases of adverse reaction due to Xuesaitong. Gansu Journal of traditional Chinese Medicine.2007;20(2):31

Liu GH, Chen LB. One case of adverse reaction caused by Xuesaitong injection. Chinese Journal of Clinical Healthcare.2006;9(6):548

Ma GX, Shi XH. One case of allergic shock caused by intravenous injection of Xuesaitong injection. Journal of Baotou Medicine.2005;29(3):15-16

Hao LZ, Wang YR. Three cases of adverse reaction due to intravenous injection of Xuesaitong injection. .Capital Medicine.1998;5(10):30

Jiang BB, Zha AQ. Report of one case of allergic shock caused by Xuesaitong injection. Qilu Pharmaceutical Affairs.2007;26(3):189

Kong XD, Guo SX, Dong YD. One case of serious adverse reaction caused by Xuesaitong injection. Medical Journal of Chinese People s' Health.2006;18(10):916

Wang J, Sun L. Three cases of adverse reaction due to Xuesaitong injection. Chinese Journal of Modern Drug Application.2008;2(24):141

Chen H, Ma JF, Wang XD. One case of serious adverse reaction caused by Xuesaitong injection. Strait Pharmaceutical Journal.2003;15(5):130

Chen RH, Lv FQ. One case of allergic shock caused by Xuesaitong injection.Chinese Remedies & Clinics.2003;3(3):213

Niu XH, Zhao D, Ren XR. One case of serious adverse reaction caused by Xuesaitong injection. Chinese Journal of Misdiagnostics.2007;7(15):3438

Yu CL. One case of serious adverse reaction caused by Xuesaitong injection. Tianjin Pharmacy.1999;11(2):57

Hu FT. Rescue and nursing of one case of delayed allergic shock caused by Xuesaitong injection. Tianjin Journal of Nursing.1997;5(2):80

Zhang XX, Zhang XY. One case of allergic shock caused by Xuesaitong injection.Northwest Pharmaceutical Journal.1998;13(5):65

Liu XT. Allergic reactions due to Xuesaitong injection. Adverse Drug Reactions Journal.2005;2:139

Xueshuantong injection No.cases4

Sun K, Liu JJ. One case of allergic shock caused by Xueshuantong injection.Clinical Focus.2011;26(10):844

Li YX. One case of allergic shock caused by Xueshuantong injection.Chinese Journal of Modern Applied Pharmacy.2008;25(4):364

Jin CL, Fang ZQ. One case of allergic shock caused by Xueshuantong injection.Chinese Journal of Misdiagnostics.2007;7(6):1420

Miao HF, Wang YJ, Ceng JF. Report of one case of allergic shock caused by Xueshuantong injection. Journal Of Practical Traditional Chinese Medicine.2006;22(9):577
